# Diurnal High Temperatures Affect the Physiological Performance and Fruit Quality of Highbush Blueberry (*Vaccinium corymbosum* L.) cv. Legacy

**DOI:** 10.3390/plants13131846

**Published:** 2024-07-04

**Authors:** Jorge González-Villagra, Kevin Ávila, Humberto A. Gajardo, León A. Bravo, Alejandra Ribera-Fonseca, Emilio Jorquera-Fontena, Gustavo Curaqueo, Cecilia Roldán, Priscilla Falquetto-Gomes, Adriano Nunes-Nesi, Marjorie M. Reyes-Díaz

**Affiliations:** 1 Departamento de Ciencias Agropecuarias y Acuícolas, Facultad de Recursos Naturales, Universidad Católica de Temuco, Temuco P.O. Box 15-D, Chile; oavila@uct.cl (K.Á.); ejorquera@uct.cl (E.J.-F.); gcuraqueo@uct.cl (G.C.); 2 Núcleo de Investigación en Producción Alimentaria, Facultad de Recursos Naturales, Universidad Católica de Temuco, Temuco P.O. Box 15-D, Chile; 3 Departamento de Ciencias Agronómicas y Recursos Naturales, Facultad de Ciencias Agropecuarias y Medioambiente, Universidad de La Frontera, Temuco P.O. Box 54-D, Chile; h.gajardo.balboa@gmail.com (H.A.G.); leon.bravo@ufrontera.cl (L.A.B.); 4 Center of Plant, Soil Interaction and Natural Resources Biotechnology, Scientific and Technological Bioresource Nucleus (BIOREN), Universidad de La Frontera, Temuco P.O. Box 54-D, Chile; alejandra.ribera@ufrontera.cl (A.R.-F.); marjorie.reyes@ufrontera.cl (M.M.R.-D.); 5 Centro de Fruticultura, Facultad de Ciencias Agropecuarias y Medioambiente, Universidad de La Frontera, Temuco P.O. Box 54-D, Chile; 6 Área de Desarrollo Rural, Instituto Nacional de Tecnología Agropecuaria, EEA INTA Bariloche, San Carlos de Bariloche 8400, Argentina; roldan.cecilia@inta.gob.ar; 7 National Institute of Science and Technology on Plant Physiology under Stress Conditions, Departamento de Biologia Vegetal, Universidade Federal de Viçosa, Viçosa 36570-900, Brazil; priscilla.gomes@ufv.br (P.F.-G.); nunesnesi@ufv.br (A.N.-N.); 8 Departamento de Ciencias Químicas y Recursos Naturales, Facultad de Ingeniería y Ciencias, Universidad de La Frontera, Temuco P.O. Box 54-D, Chile

**Keywords:** CO_2_ assimilation, leaf temperature, fruit weight, equatorial diameter, total soluble solids

## Abstract

In this study, the physiological performance and fruit quality responses of the highbush blueberry (*Vaccinium corymbosum*) cultivar Legacy to high temperatures (HTs) were evaluated in a field experiment. Three-year-old *V. corymbosum* plants were exposed to two temperature treatments between fruit load set and harvest during the 2022/2023 season: (i) ambient temperature (AT) and (ii) high temperature (HT) (5 °C ± 1 °C above ambient temperature). A chamber covered with transparent polyethylene (100 µm thick) was used to apply the HT treatment. In our study, the diurnal temperature was maintained with a difference of 5.03 °C ± 0.12 °C between the AT and HT treatments. Our findings indicated that HT significantly decreased CO_2_ assimilation (*P_n_*) by 45% and stomatal conductance (*g_s_*) by 35.2% compared to the AT treatment. By contrast, the intercellular CO_2_ concentration (Ci) showed higher levels (about 6%) in HT plants than in AT plants. Fruit quality analyses revealed that the fruit weight and equatorial diameter decreased by 39% and 13%, respectively, in the HT treatment compared to the AT treatment. By contrast, the firmness and total soluble solids (TSS) were higher in the HT treatment than in the AT treatment. Meanwhile, the titratable acidity showed no changes between temperature treatments. In our study, *P_n_* reduction could be associated with stomatal and non-stomatal limitations under HT treatment. Although these findings improve our understanding of the impact of HTs on fruit growth and quality in *V. corymbosum*, further biochemical and molecular studies are need.

## 1. Introduction

Climate change is expected to intensify extreme climate events such as infrequent and erratic precipitation, water deficit, and extreme temperatures [1,2]. In extreme temperatures, a high temperature (HT) negatively affects plant growth and crop yields [3,4,5]. Researchers have used polyethylene chambers to study the effects of HTs on crops, which is a reliable method of imposing heat treatments in field experiments [6,7,8]. It has been reported that HTs trigger morphological changes such as a reduced leaf area, shoot and root growth inhibition, leaf senescence, and sunburn on leaves and fruits [9,10,11]. At the physiological level, it has been reported that photosynthesis is the primary process affected by HT, altering Calvin–Benson cycle activity, increasing photorespiration, decreasing electron transport, photochemical reactions, and chlorophyll biosynthesis, and inducing the inactivation of Rubisco activase [12,13,14]. Otherwise, it has been reported that HTs lead to reactive oxygen species (ROS) overproduction, damaging cellular organelles and modifying structurally the thylakoid membranes in the chloroplasts at the cellular level [15,16]. However, the plant responses to HTs depends on its duration and intensity, the period of the day (day/night temperature) and the cultivar, phenological stage, and experimental conditions [15]. Currently, most studies have analyzed the responses to HTs in herbaceous annual species such as *Arabidopsis thaliana*, *Triticum aestivum*, *Oryza sativa*, *Solanum lycopersicum*, and *Solanum tuberosum* under controlled growth conditions, with significantly less information about woody species [6,9,17,18].

Highbush blueberry (*V. corymbosum* L.) is a shrub species whose fruits are characterized by their high levels of phenolic compounds and antioxidant capacity, with great human health benefits such as antidiabetic, antibacterial, anticarcinogenic, and cardiovascular protective properties [19,20,21]. Highbush blueberry production and fruit demand have increased worldwide during the last ten years [22,23]. Chile now cultivates 18,000 ha with this fruit crop, with Legacy (3217 ha), Duke (2524 ha), and Brigitta (2222 ha) being the main cultivars, established between El Maule and La Araucanía Region [24]. It has been reported that the optimal growth temperature ranges between 25 and 30 °C for *V. corymbosum* [25]. A previous study classified Bluecrop, Brigitta, Gulfcoast, and O’Neal as HT-tolerant cultivars; meanwhile, Duke and Ridge were classified as HT-sensitive cultivars, based on their morphological and physiological responses at 35/30 °C (day/night) during 90 days under greenhouse conditions [26]. On the other hand, Chen et al. [27] reported that Brigitta was the most sensitive cultivar, followed by Duke and Misty after 6 h at 35 °C under controlled conditions. In their study, Estrada et al. [28] found differential physiological and morphological responses among the Bluegold, Elliott, and Liberty cultivars when plants were exposed to temperatures 10 °C above ambient temperature. It has been reported that the fruits of *V. corymbosum* cv. Elliott can be very sensitive to HT, provoking softening, shriveling, and necrosis [29]. Thus, the responses of *V. corymbosum* to HTs might be dependent on multiple factors, including the cultivar, temperature level applied, treatment duration, phenological stage, and other experimental conditions. Although previous studies have reported the responses of some *V. corymbosum* cultivars to HTs under controlled conditions, less is documented under field conditions and no prior studies address the effects of HTs on cv. Legacy and fruit-related parameters. Therefore, this study aimed to evaluate the responses of *V. corymbosum* cv. Legacy regarding its physiological performance and fruit quality parameters to diurnal HTs under field conditions.

## 2. Results

### 2.1. Environmental Conditions during the Experiment

**Figure 1:**
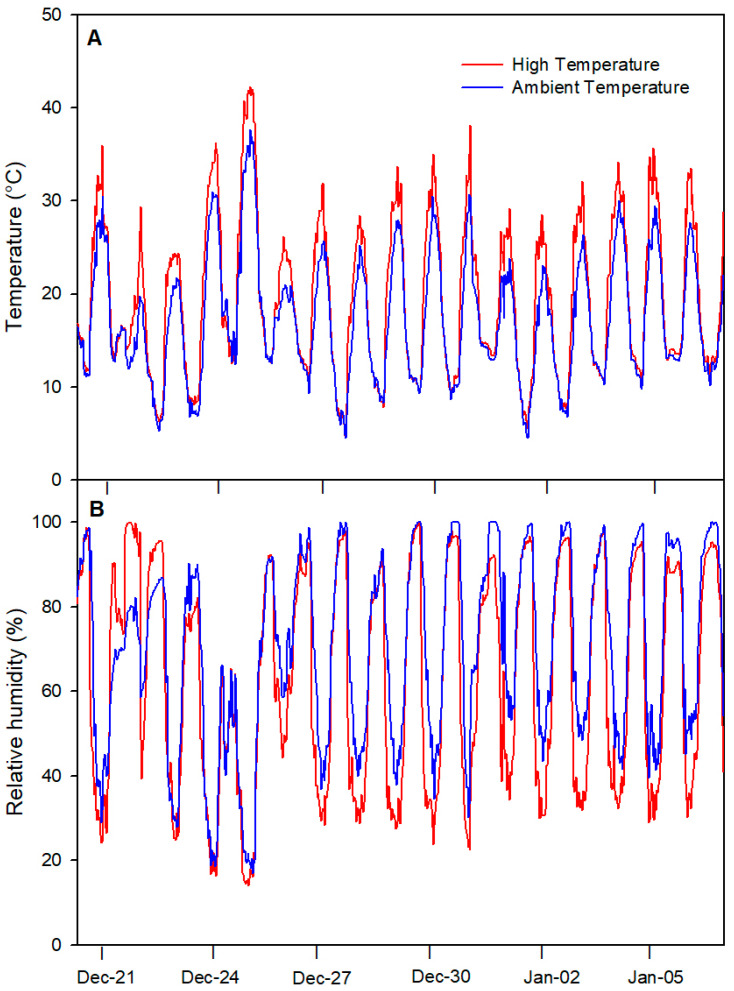
(**A**) Daily temperature (T°) and (**B**) relative humidity (RH) during the experiment: (i) ambient temperature (control) and (ii) high temperature (5 °C ± 1 °C above the ambient temperature).

Our results showed that the diurnal mean temperature (between 09:00 to 18:00 h) was 21.9 ± 0.91 °C for the ambient temperature (AT) treatment and 26.9 ± 0.91 °C for the high-temperature (HT) treatment, resulting in a significant difference of 5.03 ± 0.12 °C throughout the experiment (Figure 1A). During nighttime, non-significant changes were detected in the temperature between the AT and HT treatments. The maximum temperature registered was 37.7 °C for AT and 42.3 °C for the HT treatment on 25 December. Concerning relative humidity (HR), the diurnal mean was 55.5% for AT and 41.2% for HT (Figure 1B). The minimum RH registered was 22.5% for AT and 27.1% for HT on 25 December. During the nighttime, both treatments showed high RH values (above 95%), with no significant differences between treatments.

### 2.2. Soil Water Content, Plant Water Status, and Leaf Temperature

**Figure 2:**
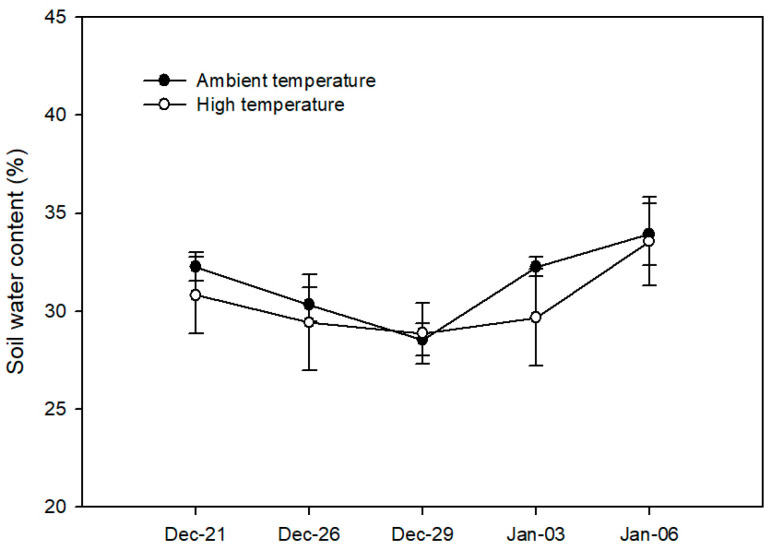
Soil water content (SWC, %) in *V. corymbosum* cv. Legacy plants subjected to two temperature treatments: (i) ambient temperature (control) and (ii) high temperature (5 °C ± 1 °C above the ambient temperature). The SWC was measured in the morning between 08:00 and 10:00 h. The value represents the means ± SE (*n* = 8).

**Figure 3:**
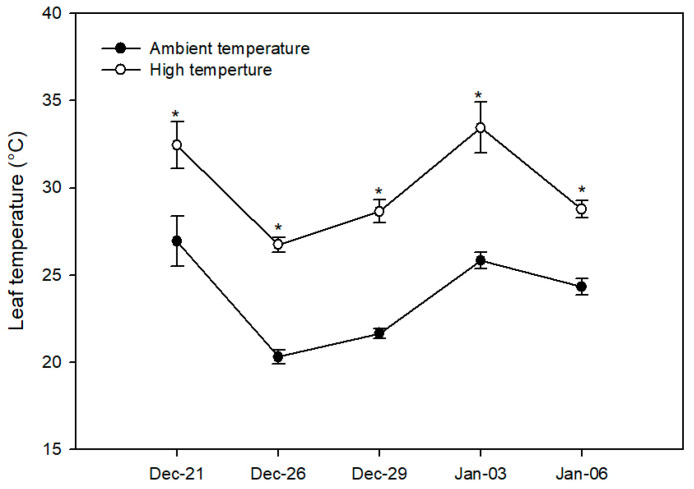
Leaf temperature in *V. corymbosum* cv. Legacy plants subjected to two temperature treatments: (i) ambient temperature (control) and (ii) high temperature (5 °C ± 1 °C above the ambient temperature). Leaf temperature was measured in the morning between 08:00 and 10:00 h. Asterisks indicate significant differences according to Student’s *t*-test (*p* ≤ 0.05). The value represents the means ± SE (*n* = 8).

Our results revealed no significant changes in the soil water content (SWC) in *V. corymbosum* plants between treatments during the experiment, showing a mean of 32.5% (Figure 2). The stem water potential (Ψ_w_), which was measured only at the end of the experiment, reached −0.32 ± 0.03 MPa for the AT treatment and −0.33 ± 0.03 MPa for the HT treatment. Regarding leaf temperature, the HT treatment led to an increase in the leaf temperature (by about 5 °C) in *V. corymbosum* plants compared to the AT treatment throughout the experiment, which was expected considering the difference in temperature between the AT and HT treatments (Figure 3). 

### 2.3. Gas-Exchange in V. corymbosum Plants

**Figure 4:**
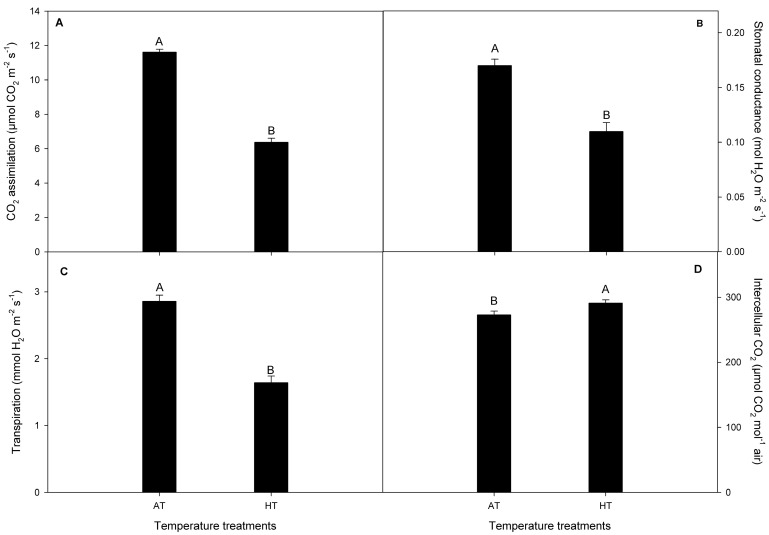
(**A**) CO_2_ assimilation (*P_n_*), (**B**) stomatal conductance (*g_s_*), (**C**) transpiration (*E*), and (**D**) intercellular CO_2_ concentration (Ci) in *V. corymbosum* cv. Legacy plants subjected to two temperature treatments: (i) ambient temperature (control) and (ii) high temperature (5 °C ± 1 °C above of ambient temperature). Different uppercase letters indicate significant differences between temperature treatments according to Student’s *t*-test (*p* ≤ 0.05). The value represents the mean ± SE (*n* = 8).

In our study, the HT treatment decreased CO_2_ assimilation (*P_n_*) by 45%, stomatal conductance (*g_s_*) by 35.2%, and transpiration (*E*) by 42% compared to the AT treatment in *V. corymbosum* plants (Figure 4). By contrast, we observed that the intercellular CO_2_ concentration (Ci) showed higher levels (about 6%) in *V. corymbosum* plants under the HT treatment than when under the AT treatment (Figure 4).

### 2.4. Fruit Quality

**Figure 5:**
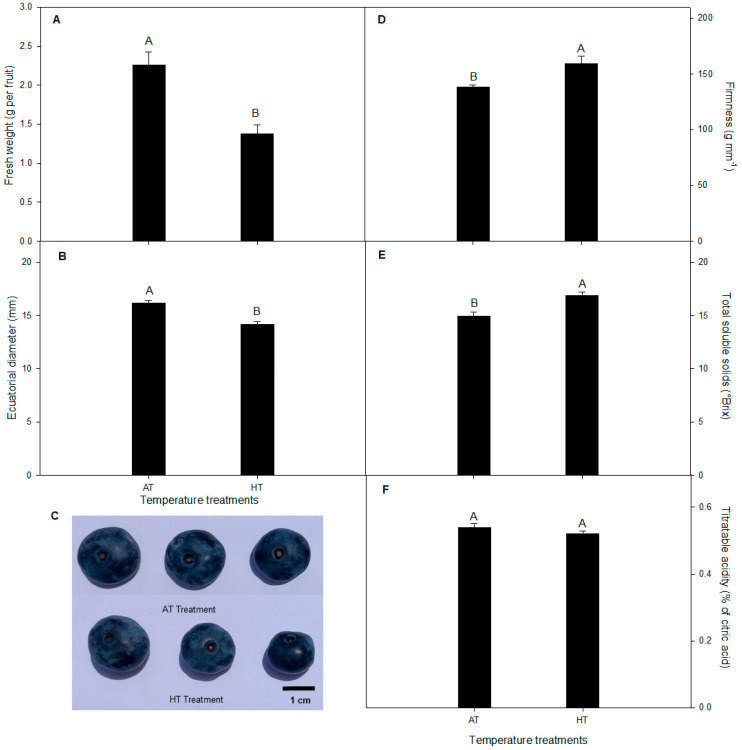
Fruit quality parameters: (**A**) fresh weight, (**B**) equatorial diameter, (**C**) fruits, (**D**) firmness, (**E**) total soluble solids, and (**F**) titratable acidity in *V. corymbosum* cv. Legacy plants subjected to two temperature treatments: (i) ambient temperature (control) and (ii) high temperature (5 °C ± 1 °C above of ambient temperature). Different uppercase letters indicate significant differences between temperature treatments according to Student’s *t*-test (*p* ≤ 0.05). The value represents the mean ± SE (*n* = 50).

Our results revealed a higher fresh weight (39%) in the fruits of *V. corymbosum* plants grown under the AT treatment than those grown under the HT treatment (Figure 5). Similarly, the equatorial diameter was increased by 13% in the fruits of plants exposed to the AT treatment (Figure 5). By contrast, the fruits of plants subjected to the HT treatment had a higher firmness and total soluble solids compared to plants grown under the AT treatment. On the other hand, the titratable acidity of fruits did not vary between the temperature treatments (Figure 5). 

### 2.5. Multivariate Analysis Based on Physiological Responses and Quality Parameters Measured in V. corymbosum under Different Treatments

**Figure 6:**
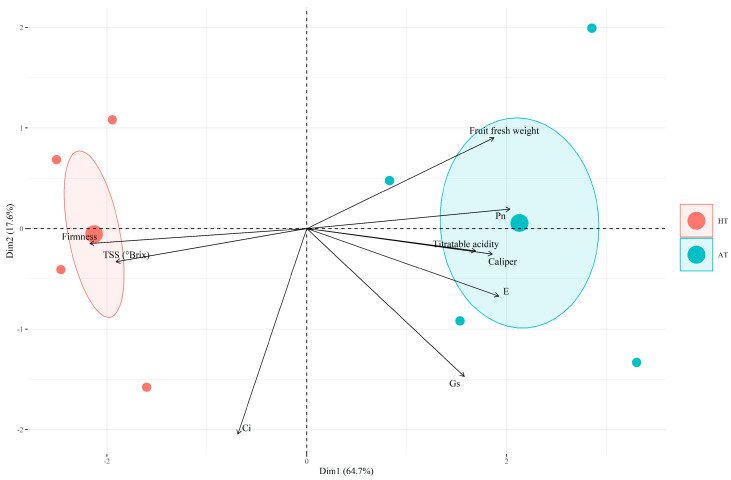
Principal component analysis (PCA) biplot of data derived from physiological responses and quality parameters measured in *V. corymbosum* fruit under different treatments: ambient temperature (AT) and high temperature (HT). All variables that are grouped are positively correlated with each other. The greater the distance between the variable and the origin, the better represented that variable is in relation to dimension 1 (first principal component) and dimension 2 (second principal component). Negatively correlated variables are displayed on opposite sides of the biplot origin. Abbreviations: CO_2_ assimilation (*P_n_*), stomatal conductance (*g_s_*), transpiration (*E*), intercellular CO_2_ concentration (Ci), and total soluble solids (TSS).

To visualize and understand the relationships between the physiological responses and quality parameters evaluated as a function of the different treatments applied, we carried out a principal component analysis (PCA) (Figure 6). The biplot shows individual replicates in each treatment combined with the measured variables. This analysis revealed a separation between the AT and HT treatments. The sum of PC1 and PC2 explained around 82.3% of the observed variability, which was responsible for separating the HT and AT treatments into distinct groups. The HT treatment positively influenced the quality parameters of firmness and TSS, but had a negative relationship with parameters such as *P_n_*, *g_s_*, *E*, and the fruit fresh weight. In general, it can be seen that HTs induce a reduction in the physiological responses analyzed, which consequently leads to a reduction in the biometric parameters of the fruit, as shown in Figure 4 and Figure 5. In addition, HTs are directly related to an increase in firmness and TSS, which can be explained by the lower fruit fresh weight found in this treatment, which implies a higher concentration of solutes in these fruits. Firmness, Ci and *g_s_* were the three characteristics that contributed most to PC1 and PC2. Titratable acidity was the characteristic that least influenced the separation of PC1 and PC2.

## 3. Discussion

### 3.1. Physiological Responses of V. corymbosum cv. Legacy Plants

High temperatures (HTs) induce several morphological and physiological changes in plants, negatively affecting crop yields [3,5]. However, less is known about the effects of HTs in *V. corymbosum* under field conditions. In this study, we evaluated the physiological and fruit quality responses of *V. corymbosum* cv. Legacy to HTs in a field experiment, using a polyethylene chamber approach to increase air temperature according to Kim et al. [7]. In our study, the HT treatment increased the diurnal temperature by 5.03 ± 0.12 °C above ambient temperature (AT) throughout the experiment (Figure 1A). Meanwhile, the soil water content (SWC) and stem water potential (Ψ_w_) were unaffected by the HT treatment in *V. corymbosum* cv. Legacy plants (Figure 2), discarding additional stressing factors in our experiment such as irrigation differences. By contrast, we found that the HT treatment increased the leaf temperature in *V. corymbosum* cv. Legacy plants, rising by 5 °C above the AT treatment (Figure 3). Concerning leaf gas exchange, *V. corymbosum* cv. Legacy plants experienced decreased *P_n_* (45%), *g_s_* (35.2%), and *E* (42%) under the HT treatment compared to the AT treatment (Figure 4). Our results agree with those of Hao et al. [26], who reported a significant decrease in *P_n_* and *g_s_* (by about 32%) in *V. corymbosum* cv. Duke subjected to HTs (35 °C), meaning that it was classified as a HT-sensitive cultivar. Meanwhile, they showed that HT-tolerant cultivars (Bluecrop, Brigitta, O’Neal, and Gulfcoast) did not exhibit significant changes in *P_n_* at high temperatures. The authors showed that HT-tolerant cultivars exhibit higher transpiration rates compared to sensitive ones, dissipating heat by leaf transpiration. They suggested that the HT tolerance might be explained by changes in stomatal traits such as the stomatal distribution, density, aperture size, and shape, allowing higher leaf transpiration to dissipate heat. However, in our study, we observed a decrease in *P_n_* and *E*, suggesting that Legacy might be classified as a sensitive cultivar. It has been widely reported that photosynthesis may be reduced by stomatal and non-stomatal limitations [30,31]. Chen et al. [32] reported that HTs negatively affect Calvin–Benson cycle enzymes such as ribulose bisphosphate regeneration (RuBP), rubisco activase (RCA), and ribulose-1,5-bisphosphate carboxylase/oxygenase (RuBisCo). Thus, in our study, the reduction in photosynthesis might be due to a negative effect on Calvin/Benson cycle enzymes. As we mentioned before, *P_n_* and *g_s_* were reduced in plants under the HT treatment. However, we also observed that *V. corymbosum* cv. Legacy plants had significantly higher Ci levels (about 6%) under the HT treatment compared to the AT treatment. Chen et al. [32] found a significant reduction in F_v_/F_m_ in four *V. corymbosum* cultivars exposed to 40 °C, indicating photoinhibitory damage under HTs, which could be associated with the reduction in *P_n_* in *V. corymbosum* cv. Legacy plants in our study. On the other hand, Hao et al. [26] reported that HT-sensitive *V. corymbosum* cvs. Duke and Blue Ridge exhibited swollen chloroplasts with significant damage, disordered grana lamella, and stromal lamella under HT treatment. In fact, Chen et al. [27] indicated that the ROS and lipid peroxidation levels increased in *V. corymbosum* plants exposed to HTs, suggesting significant damage to cellular membranes and/or thylakoid membranes. Therefore, the reduction in *P_n_* levels could be associated with stomatal and non-stomatal limitations in *V. corymbosum* cv. Legacy plants exposed to HTs in our study. 

### 3.2. Fruit Quality Changes in V. corymbosum cv. Legacy Plants

Our results revealed that the HT treatment significantly decreased the fresh weight by 39% and the equatorial diameter of fruits by 13% in *V. corymbosum* cv. Legacy compared to the AT treatment, suggesting that fruit growth was inhibited during the HT treatment. This growth inhibition could be explained by the lower *P_n_* in *V. corymbosum* cv. Legacy plants under HTs in our study (Figure 4 and Figure 5). By contrast, the firmness and total soluble solids were increased in *V. corymbosum* cv. Legacy fruits under the HT treatment compared with the AT treatment (Figure 5). Our findings agree with Bryla et al. [33], who reported that the fruit firmness and total soluble solids increase due to a reduction in fruit volume. Likewise, Faghih et al. [34] reported that smaller fruits are firmer than larger fruits due to their higher cell density. Therefore, the higher firmness and total soluble solids of fruits of plants exposed to the HT treatment could be explained by the lower fruit size (lower fruit weight and equatorial diameter) in our study. 

### 3.3. Principal Component Analysis (PCA)

The PCA shows a negative relationship between HTs and physiological responses, which was to be expected (Figure 6). It is known that at high temperatures plants tend to partially close their stomata to avoid excessive water loss through transpiration (*E*). In this context, the availability of CO_2_ can become limiting for photosynthesis, resulting in a reduction in carbohydrate production by the plant. This relationship would explain the positive correlation visible in the PCA between *P_n_*, *g_s_*, *E*, and fruit weight. The stem water potential was not altered under HTs when compared to the AT treatment at the end of the experiment. This highlights the success of this plant strategy to avoid excessive water loss under HTs. On the other hand, this generates a stomatal limitation that directly influences the final fruit production. Excessive heat can negatively affect the functioning of enzymes and degrade photosynthetic pigments and other components of the photosynthetic machinery, resulting in collateral damage to cell membranes and the inhibition of metabolic processes. Consequently, this affects the photosynthetic efficiency and reduces the biomass production of the plant. This would also explain the correlation between the physiological responses and production parameters such as the fruit fresh weight and equatorial diameter observed in the PCA.

## 4. Materials and Methods

### 4.1. Plant Material and Description of the Study Site

The field experiment was conducted at the Experimental Station of the Universidad Católica de Temuco (39°30′08″ S; 72° 47′59″ W), located in Lautaro, La Araucanía Region, Chile, during the 2022/2023 season. The plant material corresponds to three-year-old *V. corymbosum* cv. Legacy plants, which were transplanted in plastic pots containing 30 L of soil during the 2019 season. The soil was classified as the Temuco series (Andisol, Typic Hapludands) [35]. The soil texture analysis showed a silt loam surface (19.6% sand, 42.8% silt, 37.6% clay). The soil nutrient analysis showed an organic matter content of 17.21%, a pH of 5.75, P of 13 mg kg^−1^, K of 216 mg kg^−1^, Ca of 7.3 cmol+ kg^−1^, Mg of 1.47 cmol+ kg^−1^, and Na of 0.07 cmol+ kg^−1^. Agronomic management, such as irrigation, pest control, pruning, and fertilization, were performed following commercial recommendations. Weeds were manually controlled.

### 4.2. Treatments and Experimental Conditions

**Figure 7:**
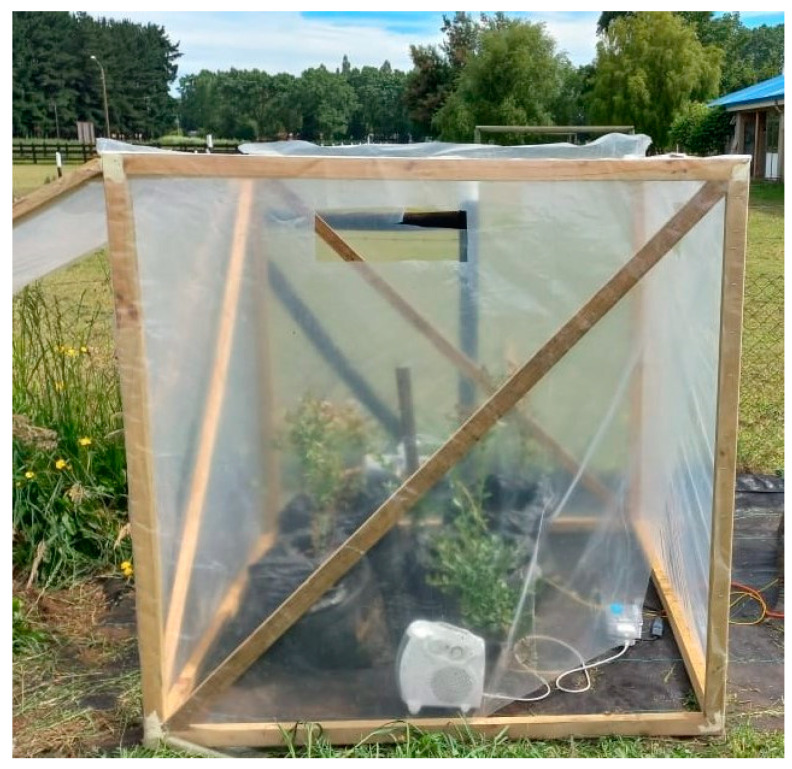
High-temperature chamber covered with transparent polyethylene (100 µm thick).

Plants were subjected to two treatments for 20 days from fruit load set (17 December) to fruit harvest (7 January): (i) ambient temperature (AT; control) and (ii) high temperature (HT; 5 °C ± 1 °C above ambient temperature). A chamber was built of wood (1.5 × 1.5 × 1.5 m) and covered with transparent polyethylene (100 µm thick) to increase the temperature in the HT treatment (Figure 7), as suggested by Ávila-Valdés et al. [6] and Kim et al. [7]. The transmittance of the polyethylene was about 95%, as measured by a Polypen (PSI, Brno, Czech Republic). The chamber was equipped with a thermostatic electric heater and controlled by a temperature regulator (Cavadevices, Buenos Aires, Argentina) [6]. To avoid extreme temperatures inside the chamber, the top was removed daily. The environmental conditions (inside and outside of chamber) were continually monitored, with the air temperature and relative humidity recorded using data loggers (Elitech Technology, Inc., San Jose, CA, USA).

### 4.3. Soil Water Content and Plant Water Status

The soil water content (SWC) was determined twice a week throughout the whole experiment using a portable time-domain reflectometer (TDR) soil moisture meter (TDR-300, Spectrum Technologies Inc., Plainfield, IL, USA). Otherwise, the plant water status was determined by measuring the stem water potential (Ψ_w_). For this, leaves were covered with aluminum foil in a plastic bag for 60 min before measurement [36]. The Ψ_w_ was determined at fruit harvest using a Scholander chamber Model 1000 (PMS, Instruments Co., Corvallis, OR, USA) between 08:00 and 10:00 h. 

### 4.4. Leaf Temperature

During the experiment, the leaf temperature was monitored twice a week using a hand-held infrared thermometer Fluke 62 Max (Fluke Corporation, Everett, WA, USA), according to Barai et al. [37]. The measurements were performed on three attached fully expanded leaves per each plant between 08:00 and 10:00 h.

### 4.5. Gas Exchange Measurement

Gas exchange was determined at the fruit harvest time using a portable infrared gas analyzer (IRGA) (Li-6400; LI-COR, Inc., Lincoln, NE, USA) following the protocol of Reyes-Díaz et al. [38]. The analyzed parameters were CO_2_ assimilation (*P_n_*), stomatal conductance (*g_s_*), transpiration (*E*), and intercellular CO_2_ concentration (Ci). The CO_2_ reference concentration was 400 μmol mol^−1^, with a flow rate of 300 mL min^−1^ and 60% relative humidity inside the leaf chamber, and the temperature was maintained at 20 ± 2 °C. The measurement was performed in vivo on attached fully expanded leaves during the light period between 08:00 and 10:00 h. Five measurements per plant were performed. 

### 4.6. Fruit Quality

When ripe (100% blue), fruits were harvested early in the morning (between 08:00 to 10:00 h), placed in 500 g clamshell containers, immediately stored in a portable refrigerator (4 °C), and transferred to the laboratory to determine the fruit quality parameters within 24 h after harvest. Fifty fruits from each plant were used to determine quality parameters such as the fresh weight (FW), equatorial diameter (ED), firmness, total soluble solids (TSS), and titratable acidity (TI). The FW was determined using a precision balance (Model BA2204B, Biobase Meihua Trading, Jinan, China). The ED and firmness were determined using a digital caliper (Mitutoyo Corp., Kawasaki, Japan) and a texture meter (FirmPro, Happyvolt, Santiago, Chile), respectively, as described by Retamal-Salgado et al. [39]. The firmness was expressed as the force in grams (g) necessary to deform the fruit in 1 mm (g mm^−1^). The total soluble solids (TSS) were determined in the fruit juice using a thermo-compensated digital refractometer (ATAGO, Mod. PAL-BX I ACID F5, Saitama, Japan) and expressed as °Brix. The titratable acidity (TA) was determined by the volumetric titration method with sodium hydroxide (0.1 N), using an automatic titrator HI-84532 (HANNA Instruments, Woonsocket, RI, USA), and expressed as the percentage (%) of citric acid, according to Mazzoni et al. [40]. For TSS and TA, 4 samples per treatment were used, each sample consisting of a juice obtained by macerating 10 fruits.

### 4.7. Experimental Design and Statistical Analysis

The experiment was performed using a completely randomized design with four replicates for each treatment. Kolmogorov–Smirnov and Levene tests were used to verify the normality of data and the variance homogeneity. A t-Student test was used to compare treatments (ambient temperature and high temperature). The statistical analyses were performed using Sigma Stat v.2.0 (SPSS, Chicago, IL, USA). The dataset was subjected to principal component analysis (PCA), preserving as much statistical information as possible. The analysis was carried out using R software version R 4.3.1 (R Core Team, Statistical computing, Vienna, Austria, 2023).

## 5. Conclusions

The negative influence of stomatal limitations on the development of *V. corymbosum* under HTs is clear, as evidenced by the physiological responses found in this study. Stomatal and non-stomatal limitations related to damage due to excessive heat certainly also have a major impact on the growth and development of fruit under HTs, but in order to really measure their contribution, more biochemical and metabolic studies are needed on *V. corymbosum* under HTs.

## Data Availability

All data supporting the findings of this study are available within the paper.
